# Does Multidisciplinary Team Simulation-Based Training Improve Obstetric Emergencies Skills?

**DOI:** 10.3390/healthcare9020170

**Published:** 2021-02-05

**Authors:** Encarna Hernández, Marcos Camacho, César Leal-Costa, María Ruzafa-Martínez, Antonio Jesús Ramos-Morcillo, Eduardo Cazorla, José Luis Díaz-Agea

**Affiliations:** 1 Faculty of Nursing, Universidad Católica de Murcia (UCAM), Av. de los Jerónimos, 135, Guadalupe, 30107 Murcia, Spain; ehsanchez@ucam.edu (E.H.); jluis@ucam.edu (J.L.D.-A.); 2 Hospital of Huercal-Overa, Av. de la Dra. Ana Parra, s/n, Huércal-Overa, 04600 Almería, Spain; 3 Faculty of Nursing, Universidad de Murcia (UM), Campus de Espinardo, 30100 Murcia, Spain; maruzafa@um.es (M.R.-M.); ajramos@um.es (A.J.R.-M.); 4 Department of Obstetrics and Gynecology, Hospital de Torrevieja, Carretera CV 95, s/n, Torrevieja, 03186 Alicante, Spain; ecazorla@torrevieja-salud.com

**Keywords:** patient simulation, obstetrics, emergencies, health team, patient safety, active learning

## Abstract

Clinical simulation in obstetrics has turned out to be a tool that can reduce the rate of perinatal morbidity and mortality. The objective of this study was to analyze the impact and evaluate the effects of training with high-fidelity simulation of obstetric emergencies on a multidisciplinary group. The quasi-experimental research study was structured in three phases: a first phase where the most important obstetric emergencies were determined, a second phase of design and development of the selected cases for simulation training, and a third and final phase where the abilities and satisfaction of the multidisciplinary team were analyzed. Three scenarios and their respective evaluation tools of obstetric emergencies were selected for simulation training: postpartum hemorrhage, shoulder dystocia, and breech delivery. The health professionals significantly improved their skills after training, and were highly satisfied with the simulation experience (*p* < 0.05). An inter-observer agreement between good and excellent reliability was obtained. Regarding conclusions, we can state that high-fidelity obstetric emergency simulation training improved the competencies of the health professionals.

## 1. Introduction

Despite the technological advances in the area of maternity, obstetric emergencies are still found in half of the maternal mortality cases in Spain, with the pregnancy, delivery, and post-partum periods being the first cause of hospital admission of women in this country [1].

The health professionals involved in the care of the newborn and the mother have the ethical and professional obligation, as well as the right to continuous training with proven efficiency, to be able to provide high-quality and excellent care [2,3]. Clinical simulation in obstetrics is an instrument, backed by evidence, that can be used for the improvement in the rate of positive perinatal results [4].

Given that a multidisciplinary team is involved in the care and attention of the patients during obstetric emergencies, it is necessary to train these professional workers as a group to guarantee excellence in their work [5].

Two of the ideal characteristics of obstetric emergency simulation is that, first, it must be ethical, and involve treating the woman and her family in these intimate and delicate times in a manner that is safe and adequate. Second, it should provide the health professionals with the possibility of repeating and practicing situations that, although not very frequent, are crucial in real life.

Training with obstetric simulations among health professionals is not infrequent outside of Spain [6,7,8], and is a standardized practice in the obstetric and gynecology departments at hospitals in many countries. However, in Spain, the centers that organize activities such as this are scarce, despite the recommendations by the WHO [9]. To conduct this study, the following objectives were set.

The general objective was to evaluate a training/intervention program in obstetric emergencies for health professionals, with the following specific objectives:

1. Identify the training needs of the health professionals associated with obstetric emergencies.
2. Design and validate a specific tool to measure the competencies in the training with obstetric simulations.
3. Design and conduct simulation scenarios in obstetric emergencies with a multi-professional group.
4. Compare the actions of the health professionals when faced with selected obstetric emergencies before and after simulation training.
5. Describe the perception and level of satisfaction of the health professionals after the obstetric simulation training.

## 2. Materials and Methods

**Figure 1:**
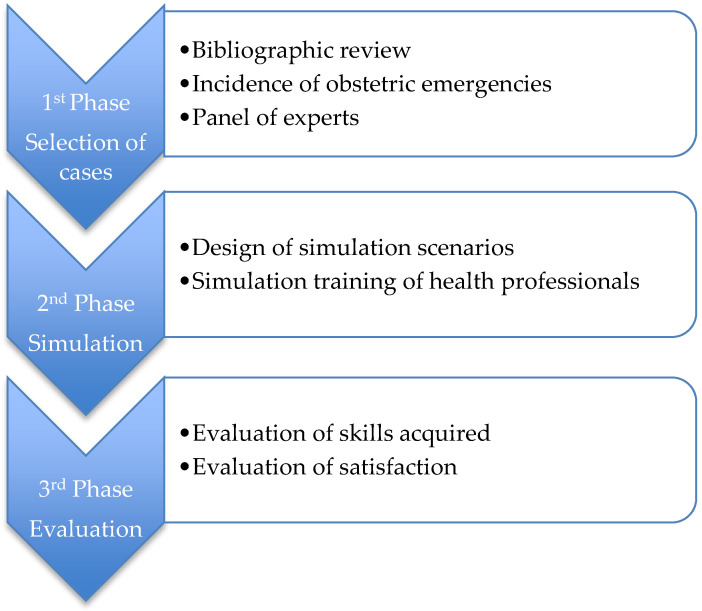
Scheme of the study phases.

In this study, the multidisciplinary training in obstetric emergencies for all obstetrics service personnel at the Hospital of Torrevieja (Spain) was proposed through a high-fidelity simulation. The research study was quasi-experimental and structured in three phases: a first phase, which consisted of the selection of cases through a bibliographic review of the incidences of obstetric emergencies in the area of study and judgement by a panel of experts. A second phase was comprised of the design of simulation scenarios and training of the health professionals on the selected obstetric emergencies, and lastly, there was a third phase, in which evaluations were conducted about the skills acquired through training with simulation and about the satisfaction of the professionals (Figure 1). This research study was conducted from April 2015 to June 2018.

### 2.1. Methodology of the First Phase

#### 2.1.1. Design of the Study and Sample

This research was a cross-sectional, observational, descriptive, and retrospective study.

First, a bibliography review was performed of the obstetric emergencies that were most recommended for training in obstetric simulation. At the same time, we sought to find the most frequent obstetric emergencies that occurred at the Hospital of Torrevieja in the period of a year (from January 2016 to December 2016), with judgement provided by a panel of experts to understand the training needs in obstetric emergencies of the health professionals who worked at the obstetrics department. As a result, ideal cases were selected for the simulation training of obstetric professionals who worked at the delivery room at the Hospital of Torrevieja.

The study population was comprised of the gynecologists, midwives, and nursing assistants who worked at the delivery room at the Hospital of Torrevieja.

- Literature Review

A literature review was conducted on the obstetric emergencies that were most recommended for training in obstetric simulation worldwide, and on the simulated obstetric cases in existing training programs in Spain.

A bibliographic search of scientific evidence was primarily performed in the following databases: Pubmed, Web of Science, EBSCOhost, Cochrane Library, and Science Direct. Other types of documents were also reviewed, such as specialized books or manuals, strategies from official organizations, legislation, protocols, or clinical practice guides. As for the strategy and search limits, the following keywords were utilized: “Simulation”, “Obstetric emergencies”, “Team-based Training”, “Shoulder dystocia”, “Post-partum hemorrhage”, “Breech Delivery”, “Eclampsia”, “Cord Prolapse”, “Maternal collapse”, and their synonyms in Spanish, in MeSH terms, along with the Boolean operators (“AND” and “OR”), which resulted in the selection of articles.

The articles selected were those in which the descriptors appeared in the title or the abstract, the search was limited to the last five years (from the year 2000), and the language was limited to Spanish or English. The search was conducted between January and May of 2016, although the search for published articles related to the research subject was continued in the years 2017 and 2018.

- Prevalence Study at the Hospital of Torrevieja

To determine the most important obstetric emergencies at the obstetrics service at the Hospital of Torrevieja for the design of scenarios that were adequate for the needs studied, a prevalence study was performed of the most frequent obstetric emergencies at the Hospital of Torrevieja in the previous twelve months (from January 2016 to December 2016).

These data were obtained thanks to the *Florence Clinico* software program, utilized to collect all of the clinical data from all of the cases handled at the hospital in the obstetrics and gynecology services, and the Department of Clinical Records, which provided the information after various joint meetings.

As search criteria, the third-trimester obstetric emergencies of pregnancy, intrapartum, and immediate postpartum were selected from all of the obstetric discharges that were handled at the hospital from January 2016 to December 2016.

- Expert Judgement

The opinions from experts was utilized due to their experience and knowledge about the most important obstetric emergencies for simulation training.

The inclusion criteria for the selection of the experts were:

- Having more than six years of work experience in obstetrics.
- Currently working at the Hospital of Torrevieja obstetrics delivery room.
- Having teaching or research experience in the field of obstetrics/nursing.
- The panel of selected experts was composed of:
- One gynecologist doctor and supervisor of the Department of Obstetrics and Gynecology at the Hospital of Torrevieja and the Hospital of the Vinalopó in Elche
- One gynecologist from the Hospital of Torrevieja, a tutor of gynecology and obstetrics residents
- One midwife supervisor from the delivery and maternity service at the Hospital of Torrevieja and the Hospital of the Vinalopó, with teaching and research experience
- One midwife, researcher, and midwife residents tutor
- Two nursing assistants and tutors of nursing assistant students

A script with questions about obstetric emergency simulations was provided, and an analysis of their answers was performed afterwards to identify areas of common agreement.

The open follow-up script included the following subjects:

- Knowledge about clinical simulation in obstetrics
- Previous participation in obstetric emergency simulations
- Interest in training on obstetric emergencies
- Needs of the multidisciplinary team in training
- Selection of cases for simulation training

#### 2.1.2. Analysis of the First Phase Data

The results were analyzed through a consensus analysis by selecting the most frequent expert answers as valid to reach a consensus.

### 2.2. Methodology of the Second Phase

#### 2.2.1. Study Design

The study utilized a quasi-experimental design, without controls and before and after, of the study units. A pre-test and post-test design was utilized, in which the health professionals were evaluated on their abilities and competencies before and after the simulation intervention.

#### 2.2.2. Sample

A non-probabilistic convenience sampling method was utilized, taking into account the list of workers who were hired by the obstetrics service. The target population was comprised of all of the health professionals (gynecologists, midwives, and nursing assistants) from the obstetrics service at the Hospital of Torrevieja, with *n* = 36 health professionals. The final sample was comprised of 30 health professionals.

#### 2.2.3. Variables

The variables that were considered in the second phase of the study are shown in the following table (Table 1).

#### 2.2.4. Data Collection

For the simulation training and evaluation of the health professionals, three different obstetrics scenarios were designed, with a form created ad hoc of each of the cases selected, and the competencies and variables differentiated and coded according to each health professional (gynecologist = 1, midwife = 2, and nursing assistant = 3). These forms are shown in detail as supplementary material (Tables S1–S3). A summary as a table is shown below (Table 2), which shows the most important aspects that served to train and evaluate the obstetric simulations.

For the postpartum hemorrhage scenario, the items from the tool validated in a study from 2016 were utilized [10]. For the case of shoulder dystocia, the items from the action protocol recommended by the SEGO (Spanish Society of Gynecology and Obstetrics) were chosen [11], as well as specific studies [12].

In the case of a breech delivery, a tool was created based on a study from 2016 [13], which validated a specific instrument for the management of this scenario, and on other studies on simulation training for a breech delivery [14].

To conduct the scenarios, a high-fidelity simulation room at the UCAM was available, as well as a control room, a debriefing and technical skills training room, and an audio-visual recording system. The high-fidelity maternal fetus simulator utilized was Lucina^®^ from CAE Fidelis.

During the training, the health professionals in groups of three (one gynecologist, one midwife, and one nursing assistant) first took part in the simulation as a team. Afterwards, in the debriefing room, the simulation was reflected upon, the SEGO and PROMPT (Practical Obstetric Multi-Professional Training) Maternity Foundation protocols were reviewed, and a questions/discussion session took place with the use of the plus/delta technique, emphasizing the correct aspects and the aspects that should be improved upon. Important issues related to team communication in obstetric emergencies were discussed, as well as the different roles and competencies of each professional, and videos were watched about the more specific techniques needed for each case. The duration of the simulation for each scenario was not longer than 10 min, and the debriefing phase lasted about 30–40 min for each scenario.

The overall time to resolution (OTR) was also evaluated in the cases of postpartum hemorrhage and shoulder dystocia before and after the simulation-based training.

### 2.3. Methodology of the Third Phase

After the debriefing, the participants took part in a second round of simulation of the cases proposed, where the acquisition of competencies was evaluated.

The evaluation of the professional competencies for each of the scenarios was done through the direct observation of the cases developed by three experts on the matter (one gynecologist, one midwife PhD, and one supervisor midwife PhD with more than eight years of professional experience), which were viewed double-blinded. To mask the viewing of the videos, a number was randomly assigned so that the experts could view them without knowing their real order.

To evaluate the quality and satisfaction of the participating health professionals, a validated satisfaction questionnaire was provided to them after finishing the obstetric emergencies simulation training. It was comprised of eight items, scored with a Likert scale with five response options [15], and was used to evaluate the aspects related to the simulation.

Accreditation was obtained for all of the health professionals who participated by the Continuous Training Commission from the Hospital of Torrevieja (HEDIMA group), with a document that certified their participation in a five-hour course.

#### Data Analysis of the Second and Third Phases

The data were collected in a Microsoft Excel^®^ sheet, and to process the information, a database was created with the SPSS© v21 software program. The descriptive statistics, such as the mean and standard deviation, were calculated, as well as frequencies and percentages according to the nature of the variable. To analyze the efficacy of the simulation program, Student’s *t*-test was utilized for related samples after verifying the assumption of the normality of the variables. To evaluate the inter-observer agreement, the intra-class correlation coefficient (ICC) was calculated [16].

### 2.4. Ethical Considerations

All participants provided a signed consent form to be videotaped and photographed for research and educational purposes. This study was evaluated and approved by the Hospital of Torrevieja ethics committee.

## 3. Results

### 3.1. Results of the First Phase

The results obtained from the bibliographic review about the recommended cases for obstetric emergencies simulation training worldwide are detailed in Table 3. It can be observed that the most common scenarios were shoulder dystocia and postpartum hemorrhage in every country, and pre-eclampsia and breech delivery in three out of four courses [17,18,19,20].

As for the simulated cases in training programs in Spain, both postpartum hemorrhage and shoulder dystocia were the most common cases in hospitals in Madrid, Santander, Barcelona, and La Coruña [21].

The results from the prevalence study of obstetric emergencies at the Hospital of Torrevieja in 2016 are shown in Table 4, with shoulder dystocia, hemorrhage, and postpartum curettage being the most common emergencies.

The results obtained from the panel of experts were delimited according to the relevance and training needs of the hospital service under study. Thus, for example, it was agreed that postpartum hemorrhage, shoulder dystocia, eclampsia, and breech delivery were obstetric emergencies where training was needed.

**Figure 2:**
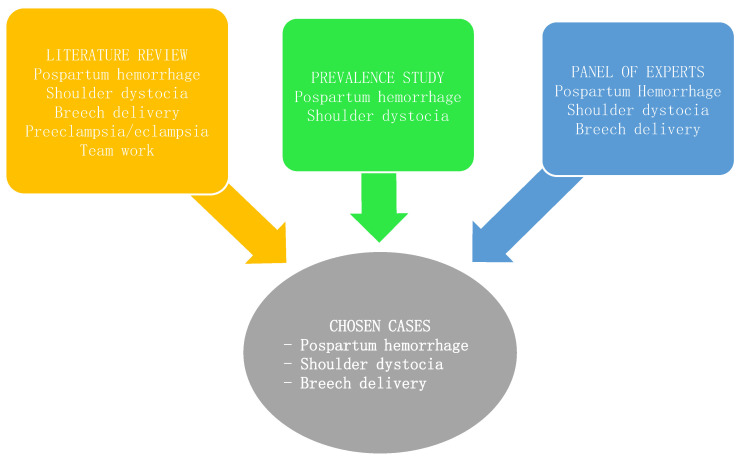
Summary of the results from the first phase.

Three cases were chosen for the design and training with simulation, with the prevalence study, the bibliographic review, and the conclusions from the consensus of the panel of experts in mind. The cases that were identified and proposed were shoulder dystocia, postpartum hemorrhage, and breech delivery (Figure 2).

### 3.2. Results of the Second Phase

#### 3.2.1. Sample Data

The total sample for this phase was composed of 30 health professionals who were 35.8 years old on average, comprised of 28 women and two men, with the following professional category distribution: seven gynecologists (23%), 17 midwives (57%), and six nursing assistants (20%).

#### 3.2.2. Form Tool

After selecting postpartum hemorrhage (PPH), shoulder dystocia (SD), and breech delivery (BD) as the simulation cases, three different forms were created that served as tools for the training and posterior evaluation of the obstetric simulation. These are shown as supplementary material (Tables S1–S3).

#### 3.2.3. The Overall Time to Resolution

**Figure 3:**
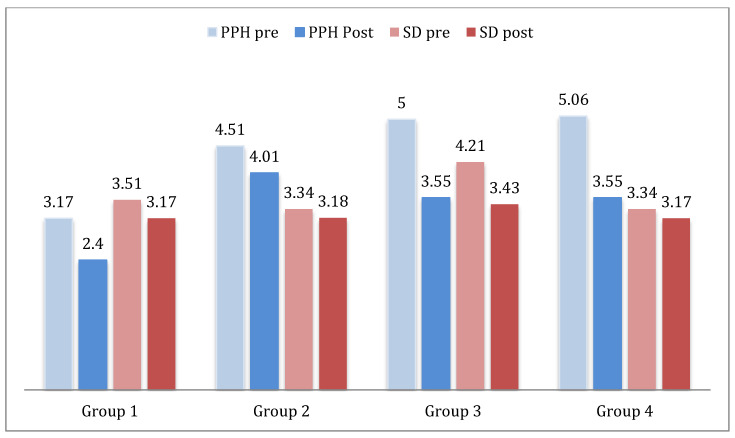
Overall time to resolution in minutes of the postpartum hemorrhage and shoulder dystocia simulations.

The overall time to resolution (OTR) of the PPH and SD scenarios in the pre-test (pre) and the post-test (post), according to the different groups of participants, are detailed in Figure 3. For the BD scenario, these data were not obtained, as it was not an obstetric emergency for which the resolution time was considered.

##### PPH: Postpartum Hemorrhage, SD: Shoulder Dystocia

In the two scenarios where the OTR was measured, PPH and SD, lower means and standard deviations were obtained after the simulation training of the case, with the results shown in Table 5.

#### 3.2.4. Inter-observer Agreement

After analyzing the intra-class correlation coefficient (ICC) results, it was observed that, in all three obstetric emergency simulation scenarios, an inter-observer agreement was obtained between moderate, good, and excellent reliability (Table 6).

### 3.3. Results of the Third Phase

#### 3.3.1. Acquisition of Competencies of the Health Professionals

- Postpartum Hemorrhage Scenario

In the PPH simulated case, the results showed that the health professionals obtained a higher mean score in the post-test than in the pre-test in all of the competencies, with these being statistically significant (*p* < 0.000) (Table 7).

The competencies that obtained the best results after the simulation training of this scenario were those that referred to the diagnosis of an emergency, C.E.8 (M = 4.66; DT = 1.09 in the pre-test and M = 6.6; DT = 0.49 in the post-test), those related to the management of the emergency itself, C.E.13 (M = 2.33; DT = 0.47 before the simulation and M = 5.13; DT = 1.16 after the training session), and the one related to adequate communication and recording of data, C.E.33 (M = 2; DT = 0.26 in the pre-test and M = 3.9; DT = 0.30 in the post-test). The competency that changed the least was C.E.14, which alludes to drug management and other specific techniques utilized in postpartum hemorrhage (M = 1; DT = 0 before training and M = 1.4; DT = 0.49 after the obstetric simulation) (Table 7).

- Shoulder Dystocia Scenario

The health professionals obtained a higher mean score in the post-test for all of the competencies that were specific to the shoulder dystocia scenario, with statistically significant differences observed (*p* < 0.05) (Table 7).

The competency related to the management of the emergency (C.E.13) obtained the greatest benefit from the simulation of this case (M = 3; DT = 0 in the pre-test and M = 6.2; DT = 0.40 in the post-test)

- Breech Delivery Scenario

The health professionals obtained a higher mean score in the post-test for all of the competencies that were specific to the breech delivery scenario, with statistically significant differences (*p* < 0.05) obtained in the competencies C.E.13, C.E.14, and C.E.33.

The competency with the highest improvement after simulation training was C.E.13, which is comprised of the techniques necessary for the management of a breech delivery (M = 2.40; DT = 0.81 in the pre-test and M = 9.73; DT = 41.88 in the post-test). The competency that was least affected after the simulation was C.E.8, related to specific techniques performed only by the gynecologist, such as the use of Piper forceps for the delivery of the aftercoming head (M = 2.83; DT = 0.379 in the pre-test and M = 2.86; DT = 1.0 in the post-test).

#### 3.3.2. Satisfaction of the Health Professionals

The health professionals who finished the simulation course completed the questionnaires, with a response rate of 100%. The answers obtained are shown in Table 8.

In 100% of the items, level four (in agreement) and level five (in complete agreement) responses were obtained, indicating the great similarity of the responses from all of the participants, and an SD (standard deviation) with little variation at both ends.

The mean score for all of the items was 4.9 (SD = 0.1), except for item one (the scenarios were real), which obtained a lower mean, although it was still high (M = 4.7; SD = 0.4).

After an analysis according to item was performed, it was observed that 29 (96.66%) out of the 30 participants scored five out of the eight the items (items three, four, six, seven, and eight) of the questionnaire with a five (in complete agreement), with only one participant (3.33%) scoring these items with a four (in agreement).

## 4. Discussion

### 4.1. Discussion of the First and Second Phases

When facing a postpartum hemorrhage, the time it takes to act and control the emergency is vital for reducing the mother’s morbidity and obtaining optimal maternal and fetal results [17], and this is possible thanks to simulation training [18]. In our simulation, the results showed that all of the competencies evaluated obtained statistically significant improvements. The overall time to resolution decreased after simulation training, so that the health professionals were able to train on and improve the response time when facing this obstetric emergency.

Teamwork and the coordination of tasks form part of the activities of this competency (S.C.13), which improved after the simulation, and which are fundamental for the good functioning of the medical team in any obstetric emergency [19].

A recent systematic review [7] in which 233 articles were reviewed concluded that the introduction of training in multidisciplinary teamwork in a simulation-based environment prevents mistakes during practice, improving patient results. In postpartum hemorrhage, the visual estimation of bleeding and the ability to rapidly recognize the gravity of the situation are also important, with these competencies improving after the simulation. This evidence demonstrates how a sub-estimation of the bleeding is one of the most-common errors facing a real-life obstetric emergency [20].

In general terms, the main failure of health professionals when faced with shoulder dystocia is based on the inadequate documentation of the maneuvers performed [20]. In the shoulder dystocia simulation scenario, all of the competencies achieved a statistically significant increase, especially S.C.13 and S.C.33, the competencies related to the management of the dystocia, teamwork, and communication skills. It should be highlighted that, within these competencies, we find “time counting”, which includes annotating the time when the head exits, counting the seconds after the resolution of the shoulder dystocia maneuver, and counting the minutes until the expulsion of the fetus, as recommended by the SEGO in its action protocol for shoulder dystocia [22].

Facilitating the health professionals, who at present are not used to performing vaginal breech delivery, with the opportunity to practice this almost-abandoned technique will perhaps help in decreasing the growing number of caesarean sections planned due to breech delivery cases, with a subsequent decrease in maternal and fetal morbidity [23]. In the breech delivery scenario, the results of the competencies evaluated showed statistically significant increases after the simulation, especially highlighting the S.C.14 competency, which deals with the management and skills used for the different aid techniques when performing a vaginal breech delivery.

In a quasi-experimental study in 2019 in Saudi Arabia [8], nurses and midwives were trained on postpartum hemorrhage, shoulder dystocia, and uterine inversion. The results concluded that the simulation increased the knowledge and competencies of the health professionals.

### 4.2. Discussion of the Third Phase

The results obtained from the satisfaction questionnaire qualified the simulation training experience as very positive. Most of the participants indicated that the clinical simulation experience helped them to develop critical thinking and decision-making skills in obstetric emergencies and improve their technical and non-technical abilities. This has also been shown in diverse studies, which showed that learning through clinical simulation was not only a way to train and to delve into the technical aspects of the competencies, but the non-technical aspects as well, such as communication skills, teamwork, leadership, and following protocols [15,19,24,25].

Communication skills in obstetric emergencies are necessary aspects that must be trained on to obtain better responses to these events [2]. The results of our study showed that, after simulation training, the trained health professionals obtained a higher response index (in S.C.33 of the three planned scenarios), and increased their ability to respond when informing the patient, the team, and family members. Other studies have also provided evidence for this [26].

The course participants who took part in the debriefing session after the simulation scenario indicated learning from their own experience and from the experiences of others as well. After watching the recorded session on the screen, they were able to analyze the strong points and the aspects that should be improved, and they were also able to review the action protocols and the roles of each of the health professionals by commenting on the actions taken as a group, at the same time promoting learning and group interaction. In the bibliography, we also found authors who argued that debriefing, as a fundamental part of the clinical simulation learning methodology, allows the participants to talk about what had occurred, discuss, reflect, analyze, and establish a critical judgement after taking part in the staged case [27,28].

As possible limitations, the resulting post-training assessment was limited to being carried out at the end of the simulation training, and an evaluation of the knowledge, skills, and repetition of the simulation training is necessary months after to reinforce knowledge and improve technical skills practice. Another limitation was the convenience sampling, which would condition the generalization of the results to the general population, as well as the risk of bias associated with this type of sample.

## 5. Implications

The participants in our study, who for the most part did not have a previous experience with high-fidelity simulation, evaluated the site and availability of the simulation installations where the obstetric emergency course took place at the UCAM as very positive due to their advanced level of technology, the high-quality resources, and ideal organization, as these are not available at the Hospital of Torrevieja.

Although we must highlight that clinical simulation does not try to supplant the training in situ that health professionals receive at work, it can be used to complement this training, to comprehensively obtain the knowledge and skills necessary, and to cater to the training needs of health professionals. The creation of a clinical simulation unit at the hospital itself and the mandatory and annual programming of multidisciplinary obstetric simulation courses are some of the options that could be planned as a future line of improvement of the quality of care and safety of the patient.

## 6. Conclusions

High-fidelity obstetric emergency simulation training improved the competencies of health professionals in a safe and effective manner, allowing for the practice of not-so-frequent events without any risk to the patient. In our study, the most important training needs in obstetric emergencies of the health professionals were postpartum hemorrhage, shoulder dystocia, and breech delivery.

The tool created for the evaluation of the competencies of professionals related to obstetric emergencies obtained an adequate inter-observer reliability and content validity.

The design of the scenarios, based on the learning objectives detected from the health professionals’ needs, allowed for a structured and safe obstetric simulation training. The level of the competencies of the health professionals significantly increased after the intervention, and the participants were very satisfied with the experience.

## Figures and Tables

**Table 1:** Variables of the second phase of the study.

Variables 2nd Phase		
Independent var.	Dependent var.	
Age	Non-technical	Technical
Sex	Coordination of tasks	39 variables Techniques from the three specific scenarios
Profession	Working in teams	
Previous simulation experience	Communication with patient	
Experience in obstetrics	Communication with team	
Nationality	Communication with family	
Observer	Perception of professionals	

**Table 2:** Summary of the form for the training and evaluation of the simulation.

Name of scenario	Obstetric Emergency to Simulate
Patient data	Relevant data
	Health status of the pregnant woman
	Summary of the scenario
Objectives to be met in the simulation	Evaluation of the patient
	Activities/techniques to be performed
	Coordination, teamwork
Professional involved	Gynecologist, midwife, nursing assistant
OTR	Overall resolution time of the emergency
S.C.	Specific competencies to be trained on/evaluated in each scenario

OTR: Overall time to resolution of the case; S.C.: specific competency for nursing/medicine.

**Table 3:** Recommended cases for the simulation training of obstetric emergencies worldwide.

Country	UK, New Zealand, and Australia	Latin America, Africa, and India	United States	Canada
Course	PROMPT	PRONTO	ECO	ALARM
Obstetric cases	Shoulder dystocia	Shoulder dystocia	Shoulder dystocia	Shoulder dystocia
	Severe pre-eclampsia and eclampsia	Postpartum hemorrhage	Postpartum hemorrhage	Pre-eclampsia and eclampsia
	Postpartum hemorrhage	Pre-eclampsia and eclampsia	Cord prolapse	Postpartum hemorrhage
	Breech delivery	Neonatal resuscitation	Breech delivery	Breech delivery
	Uterine inversion	Choriamnionitis		Maternal sepsis
	Cord prolapse	Teamwork		Instrumental delivery
	Maternal sepsis	Communication		
	CPR			
	Maternal anesthetic emergencies			
	Intrapartum fetal monitoring			
	Teamwork			
	Caregiving to the second twin			
	Neonatal resuscitation			

**Table 4:** Obstetric emergencies at the Hospital of Torrevieja in 2016.

Obstetric Emergency	Number of Cases (2016)
Eclampsia	1
Shoulder dystocia	6
Postpartum hemorrhage	11
Obstetric trauma (third and fourth degree tears)	7
Emergency obstetric hysterectomy	0
Uterine rupture	0
Postpartum curettage	11
Postpartum thromboembolic disease	0
Blood or blood products transfusion	4

**Table 5:** The mean (M) and standard deviation (SD) of the OTR from the postpartum hemorrhage and shoulder dystocia scenarios pre- and post-training.

OTR	Pre	Post	Pre-Post	
	M/SD	M/SD	*t*-Test (gl)	*p*
PPH	4.48/0.70	3.50/0.52	11.24 (29)	0.000
SD	3.57/0.33	3.22/0.10	8.12 (29)	0.000

PPH: Postpartum hemorrhage, SD: shoulder dystocia, OTR: overall time to resolution.

**Table 6:** Intra-class correlation coefficient for the different scenarios.

	Pre-Test				Postest			
	ICC	Confidence Interval 95%		*p*	ICC	Confidence Interval 95%		*p*
**PPH**		Lower	Upper			Lower	Upper	
S.C.8	0.981	0.966	0.990	0.000	0.700	0.449	0.847	0.000
S.C.13	0.549	0.173	0.770	0.005	0.970	0.944	0.985	0.000
S.C.14	0.865	0.753	0.931	0.000	0.900	0.817	0.949	0.000
S.C.33	0.777	0.592	0.887	0.000	0.833	0.693	0.915	0.000
**DH**								
S.C.8	0.745	0.533	0.870	0.000	0.808	0.649	0.902	0.000
S.C.13	0.600	0.267	0.796	0.001	0.917	0.849	0.958	0.000
S.C.33	0.812	0.654	0.904	0.000	0.574	0.219	0.783	0.003
**BD**								
S.C.8	0.875	0.771	0.936	0.000	0.819	0.669	0.908	0.000
S.C.13	0.568	0.208	0.780	0.003	0.643	0.345	0.818	0.000
S.C.14	0.921	0.854	0.960	0.000	0.844	0.714	0.920	0.000
S.C.33	0.819	0.668	0.908	0.000	0.810	0.652	0.903	0.000

S.C.: Specific competency; ICC: intra-class correlation coefficient; PPT: postpartum hemorrhage; SD: shoulder dystocia; BD: breech delivery.

**Table 7:** Differences in means (M) between the pre-test and post-test results obtained in the scenarios.

	Pre-Test		Postest		Pre-Test/Postest	
**PPT**	M	SD	M	SD	t(gl)	*p*
Diagnosis (S.C.8)	4.66	1.09	6.6	0.49	15.314 (29)	0.000
Management (S.C.13)	2.33	0.47	5.13	1.16	15.38 (29)	0.000
Drugs (S.C.14)	1	0	1.4	0.49	4.39 (29)	0.000
Recording and communication (S.C.33)	2	0.26	3.9	0.30	34.10 (29)	0.000
**SD**						
Diagnosis (C.E.8)	3.26	0.44	3.46	0.50	2.69 (29)	0.012
Management (C.E.13)	3	0	6.2	0.40	43.08 (29)	0.000
Recording and communication (S.C.33)	2.06	2.06	3.93	3.93	23.54 (29)	0.000
**BD**						
Diagnosis (S.C.8)	2.83	0.379	2.866	0.345	1.0 (29)	0.326
Management (S.C.13)	2.40	0.813	9.733	0.691	41.88 (29)	0.000
Specific techniques (S.C.14)	1.00	0.262	1.400	0.498	3.89 (29)	0.001
Recording and communication (S.C.33)	2.50	0.508	2.700	0.466	2.69 (29)	0.012

S.C.: Specific competency; PPT: postpartum hemorrhage; SD: shoulder dystocia; BD: breech delivery.

**Table 8:** Descriptive statistics of the satisfaction questionnaire.

ITEMS		*n (%)*					M (SD)
		1	2	3	4	5	
1.	The scenarios were real	0 (0%)	0 (0%)	0 (0%)	7 (23.33%)	23 (76.66%)	4.7 (0.4)
2.	The experience has improved my technical skills	0 (0%)	0 (0%)	0 (0%)	2 (6.66%)	28 (93.33%)	4.9 (0.2)
3.	It helps with the development of critical thinking and decision making	0 (0%)	0 (0%)	0 (0%)	1 (3.33%)	29 (96.66%)	4.9 (0.1)
4.	The prioritization skills taught using simulation are valuable	0 (0%)	0 (0%)	0 (0%)	1 (3.33%)	29 (96.66%)	4.9 (0.1)
5.	The experience with the simulation has increased my security	0 (0%)	0 (0%)	0 (0%)	2 (6.66%)	28 (93.33%)	4.9 (0.2)
6.	The interaction with the simulation has improved my clinical competence	0 (0%)	0 (0%)	0 (0%)	1 (3.33%)	29 (96.66%)	4.9 (0.1)
7.	The simulation has helped me to integrate theory and practice	0 (0%)	0 (0%)	0 (0%)	1 (3.33%)	29 (96.66%)	4.9 (0.1)
8.	In general, the experience of working with clinical simulation has been satisfactory	0 (0%)	0 (0%)	0 (0%)	1 (3.33%)	29 (96.66%)	4.9 (0.1)

## Data Availability

The data presented in this study are available on request from the corresponding author. The data are not publicly available due there are unpublished results.

## Supplementary Materials

The following are available online at https://www.mdpi.com/2227-9032/9/2/170/s1. Table S1, Form for the training and evaluation of the postpartum hemorrhage simulation; Table S2, Form for the training and evaluation of the Shoulder Dystocia; Table S3, Form for the training and evaluation of a Breech Delivery.
