# Supplementary material for: Does Multidisciplinary Team Simulation-Based Training Improve Obstetric Emergencies Skills?
Source: Healthcare (Basel). 2021 Feb 5;9(2):170. doi: 10.3390/healthcare9020170 (PMC7915121; doi:10.3390/healthcare9020170)
Supplement: Supplementary file 1 [file healthcare-09-00170-s001.pdf]

healthcare MDPI

Supplementary material

Table S1. Form for the training and evaluation of the postpartum hemorrhage simulation.

Clinical simulation scenario: Postpartum hemorrhage

Sex:

Age:

Nationality:

Profession:

Experience with simulation? Yes / No

Years of experience in your profession:

Observer: 1 2 3

Patient data:

Woman, postpartum (G3P3), has been attended during normal vaginal delivery in the delivery room

Normal pregnancy

Penicillin allergy

Without personal or surgical antecedents of interest

Scenario:

The patient is in the delivery room. She has had a rushed delivery lasting 2 hours, and 25 minutes after, after the physiological delivery, show abundant bleeding. She has a 14G peripheral venous catheter. She is accompanied by her husband and her baby in arms with skin-to-skin contact. The midwife and the assistant nurse have provided assistance in the delivery, and after evaluating the first signs of the abundant bleeding, alert the gynecologist on call. The hospital admittance analysis shows normal values.

Objectives:

1. Assessment of the possible causes of the postpartum hemorrhage (tone, trauma, thrombin, tissue)
2. Volemic repositioning through the fast infusion of fluids through two large gauge peripheral venous accesses
3. Guarantee tissue oxygenation, administer oxygen, extract blood and ask for crossmatch tests, deal with hemostasis alterations and at the same time identify and control the cause of the hemorrhage.

Perform specific maneuvers, such as a fundal massage, ask for an echography to rule out other, assess the Bakri balloon as a treatment for uterine atony (oxytocin, ergot alkaloids such as ergonovine (Ergotrate) or methylergonovine (Metherghin) or prostaglandins.

4. Teamwork
5. Ask for help soon and alert the rest of the team (obstetrician and anesthesiologist), activate the blood bank and alert the hematologist.
6. Coordination and distribution of tasks

COMPETENCY	OBJECTIVES	ACTIVITIES	YES	NO	OBSERV.
**C.E.8** **Ability to recognize and interpret normal or changing signals of normal/bad health, suffering, inability (assessment and diagnosis)**	Evaluation of the patient and scenario planned (basic measures and search for etiology)	Measure vital signs (2,3)[1]			
		Evaluate the causes of PPH (1,2)			
		Evaluate medical history (1,2)			
	Recognizing obstetric emergency	Ask for help (2,3)			
		Extract blood (2)			
		Ask for crossmatch tests, if needed (1,2)			
		Ask for blood (1,2)			
		Prepare the equipment needed (2,3)			
**C.E.13** **Ability to put into practice health and safety principles, including basic first aid and emergency procedures**	Start the emergency measures	Uterine massage (1,2)			
		Place a large gauge peripheral catheter, if necessary (2)			
		Volemic repositioning (2,3)			
		Administration of O_2 (2,3)			
		Vesical catheter (1,2)			
		Coordination of tasks (1,2,3)			
		Teamwork (1,2,3)			
**C.E.14** **Ability to safely administer drugs and provide other therapies**	Has knowledge about drugs	Adequate administration of uterotonics and/or prostaglandins (1,2)			
	Has knowledge about specific techniques (gynecologist)	Fundal massage of the uterine wall (1)			
		Ask for echography to discard other causes (1)			
		Take to surgery room for curettage or Bakri balloon (if necessary) (1)			
**C.E.33** **Ability of inform, record, document and derive care using the appropriate technologies**	Utilizes adequate nurse/doctor language	Communication with the patient (1,2,3)			
		Communication with the team (1,2,3)			
		Communication with the family members (1,2,3)			
		Estimation of bleeding (1,2,3)			
**Overall time to resolution**					**mm:ss**

Table S2. Form for the training and evaluation of the Shoulder Dystocia.

Clinical simulation scenario: Shoulder Dystocia

Sex:

Age:

Nationality:

Profession:

Experience with simulation? Yes / No

Years of experience in your profession:

Observer: 1 2 3

Patient data:

Woman, primigravida (G1P0), 41+5 weeks of pregnancy, is found in the stage of expulsion with a shoulder dystocia diagnosis

Scenario:

The pregnant woman is on the parturition bed in lithotomy position, accompanied by her partner, the midwife and the assistant nurse. We find ourselves with a partial extraction of the fetus head, turtle sign, and no external rotation of the head is produced.

Spontaneous birth with SROM> 12 hours of normal evolution

EFW: 3700 g

Maternal obesity. BMI>25

NKDA.

Objectives:

1. Assess and identify signs of shoulder dystocia
2. Alert the team and ask for help.
3. Ask the woman not to push and avoid the Kristeller maneuver.
4. Perform the McRoberts maneuver and apply suprapubic pressure adequately.
5. Assess the need to perform an episiotomy.
6. Assessment of the posterior shoulder and internal maneuvers (Rubin II-Woods and Jacquemier or Barnum).
7. Assess the probability of performing the Gaskin maneuver.
8. Assess third degree maneuvers such as fracture of the fetal clavicle, the Zavanelli maneuver, symphysiotomy, and/or hysterectomy.
9. Teamwork, coordination of tasks.

**COMPETENCY**	**OBJECTIVES**	**ACTIVITIES**	**YES**	**NO**	**OBSERV.**
**C.E.8** **Ability to recognize and interpret normal or changing signals of normal/bad health, suffering, inability (assessment and diagnosis)**	Evaluation of the patient and scenario planned (basic measures and search for etiology)	Recognize signs of shoulder dystocia (1,2,3)			
		Evaluate medical history (1,2,3)			
		3. Ask the woman not to push (1,2,3)			
		Avoid Kristeller maneuver and traction of the fetal head (1,2)			
	Recognizing obstetric emergency	Ask for help (2,3)			
		Prepare the equipment needed (2,3)			
**C.E.13** **Ability to put into practice health and safety principles, including basic first aid and emergency procedures**	Start the emergency measures	McRoberts maneuver (1,2)			
		Suprapubic pressure (1,2)			
		Consider episiotomy(1,2)			
		Gaskin maneuver (if needed) (1,2)			
		Extraction of the posterior head and/or internal maneuvers (1,2)			
		Performing third-degree maneuvers (if necessary) (1)			
		Coordination of tasks (1,2,3)			
		Teamwork (1,2,3)			
**C.E.33** **Ability of inform, register, record and derive care using the appropriate technologies**	Utilizes adequate nurse/doctor language	Communication with the patient (1,2,3)			
		Communication with the team (1,2,3)			
		Communication with the family members (1,2,3)			
		Gas measurements.- pH (1,2,3)			
		Time calling (1,2,3)			
**Overall time to resolution**					**mm:ss**

Table S3. Form for the training and evaluation of a Breech Delivery.

Clinical simulation scenario: Shoulder Dystocia

Sex:

Age:

Nationality:

Profession:

Experience with simulation? Yes / No

Years of experience in your profession:

Observer: 1 2 3

Patient data:

Expectant female (G2P1) with 38 weeks of pregnancy arrives at the emergency obstetrics service in stage of expulsion and breech presentation.

Scenario:

The woman is in the delivery room, in stage of expulsion with voluntary pushing. After examination: VE: Complete dilatation, Hodge I plane, SROM: clear liquid. Breech presentation. FRH: 140 bpm

Normal pregnancy. NKDA. Without personal or surgical antecedents of interest

EFW: 3100 g

Objectives:

1. Understand and perform the necessary maneuvers for assisting in a vaginal breech delivery in a safe manner.
2. Avoid unnecessary interventions such as excessive traction of the fetus' legs and body.
3. Continuous monitoring of the fetus during the delivery process.
4. Offer and provide anesthetics to the mother if she wishes as much as possible.
5. Adequately inform the mother and companion about the delivery process and evolutions.
6. Provide an environment of teamwork and coordination.
7. Adequately record all the data in the medical history.

COMPETENCY	OBJECTIVES	ACTIVITIES	YES	NO	OBSERV.
**C.E.8** **Ability to recognize and interpret normal or changing signals of normal/bad health, suffering, inability (assessment and diagnosis)**	Recognizing obstetric emergency	Perform vaginal examination (1,2)			
		Ask for help (2,3)			
		Prepare the equipment needed (2,3)			
**C.E.13** **Ability to put into practice health and safety principles, including basic first aid and emergency procedures**	Attention during the stage of expulsion (Rojas-Mauriceau maneuvers) (1,2)	Assess episiotonomy			
		Encourage pushing (1,2,3)			
		Coordination of tasks (1,2,3)			
		Allow the lowering of the legs without traction			
		Adequate extraction of the legs			
		Adequate placement of hands on the body of the fetus			
		Gentle downward traction of the fetus body			
		Adequate rotation of the baby for assisting in the extraction of the arms			
		Adequate extraction of the arms			
		Extraction of the head with Mauriceau maneuver			
**C.E.14** **Ability to safely administer drugs and provide other therapies**	Has knowledge about specific techniques (gynecologist)	Fetal extraction according to Bracht maneuver (as a unit and in hyperextension) (1,2)			
		Final extraction of the head using Forceps (1)			
**C.E.33** **Ability of inform, register, record and derive care using the appropriate technologies**	Utilizes adequate nurse/doctor language	Communication with the patient (1,2,3)			
		Communication with the team (1,2,3)			
		Communication with the family members (1,2,3)			
